# Personality Disorders in Older Adults: a Review of Epidemiology, Assessment, and Treatment

**DOI:** 10.1007/s11920-020-1133-x

**Published:** 2020-02-06

**Authors:** Krystle A.P. Penders, Inge G.P. Peeters, Job F.M. Metsemakers, Sebastiaan P.J. van Alphen

**Affiliations:** 1 Department of Family Medicine, School CAPHRI, Care and Public Health Research Institute, Maastricht University (UM), P.O. box 616, 6200 MD Maastricht, The Netherlands; 2 Department of Treatment and Guidance, Envida, Maastricht, The Netherlands; 3 Department of Integrated Care, Maastricht University Medical Centre + (MUMC+), Maastricht, The Netherlands; 4 Faculty of Psychology & Educational Sciences, Department of Psychology, Vrije Universiteit Brussel (VUB), Brussels, Belgium; 5 Department of Old Age Psychiatry, Mondriaan Hospital, Heerlen-Maastricht, The Netherlands; 6 School of Social and Behavioral Sciences, Department of Medical and Clinical Psychology, Tilburg University, Tilburg, The Netherlands

**Keywords:** Personality disorder(s), Older adults, Elderly, Epidemiology, Assessment, Treatment

## Abstract

**Purpose of Review:**

The aim of the paper is reviewing recent literature on the epidemiology, assessment, and treatment of personality disorders (PDs) among older adults (≥ 60 years).

**Recent Findings:**

Since 2015, 12 primary empirical studies have been published addressing PDs in older adults; 3 addressing epidemiological aspects, 6 on assessment, 2 exploring both epidemiology and assessment, and 1 examining treatment. PD research in older adults is steadily growing and is predominantly focused on assessment. The studies showed that PDs were rather prevalent ranging from 10.6–14.5% in community-dwelling older adults, to 57.8% in nursing home–residing older adults. The Severity Indices of Personality Problems-Short Form, Gerontological Personality disorders Scale, and Assessment of DSM-IV Personality Disorders turned out to be promising instruments for assessing PDs in later life. Furthermore, schema therapy seems to be a feasible and effective intervention.

**Summary:**

Despite promising findings, there is an urgent need for studies addressing PDs in older adults, especially studies investigating epidemiological aspects and treatment options. Furthermore, new areas of interest arise such as PDs in other settings, and behavioral counseling.

## Introduction

In 2000, Agronin and Maletta highlighted the research gap on personality disorders (PDs) in older adults, suggesting that the lack of longitudinal data and the absence of age-appropriate diagnostic instruments as well as an elderly adjusted nosology contributed to this dearth of research [1]. Fifteen years later, van Alphen and colleagues reviewed the scientific data and concluded that still only a modest number of studies have focused on PDs in later life. However, empirical research within this field did increasingly address the absence of appropriate assessment options by focusing on psychometric properties of age-specific personality tests, the age-neutrality of instruments, and validation of personality inventories in older adults. Treatment studies were nevertheless scarce [2•].

Meanwhile, the nosological difficulties remained. Although the age-appropriateness of the PD criteria was increasingly debated (e.g., [3]), the upcoming editions of the DSM [4, 5] are inattentive to PDs in later life nor age-related aspects. As van Alphen and colleagues [2•] discussed the role of the current classification system of PDs in later life, they also saw opportunities which the recent included alternative dimensional model might have in stimulating PD research in older adults.

PDs in older adults are quite prevalent and given the aging population, clinicians are often confronted with these older adults. Considering the serious consequences, there is a clinical need for adequate assessment and treatment options for PDs in older adults. To keep track on recent developments and research initiatives which may provide considerations for clinical practice, this article evaluates primary studies on the epidemiological, diagnostic, and treatment aspects of PDs in older adults of the last 6 years.

## Methods

A literature search was performed between November 2018 and April 2019, using the databases of Medline/Pubmed and PsycINFO, to review papers published between July 2014 and April 2019, describing the epidemiology, assessment, and treatment of personality disorders (PDs) in older adults (≥ 60 years). This interval was chosen in order to include studies published after the most recent literature review on a similar subject matter [2•].

**Fig. 1:**
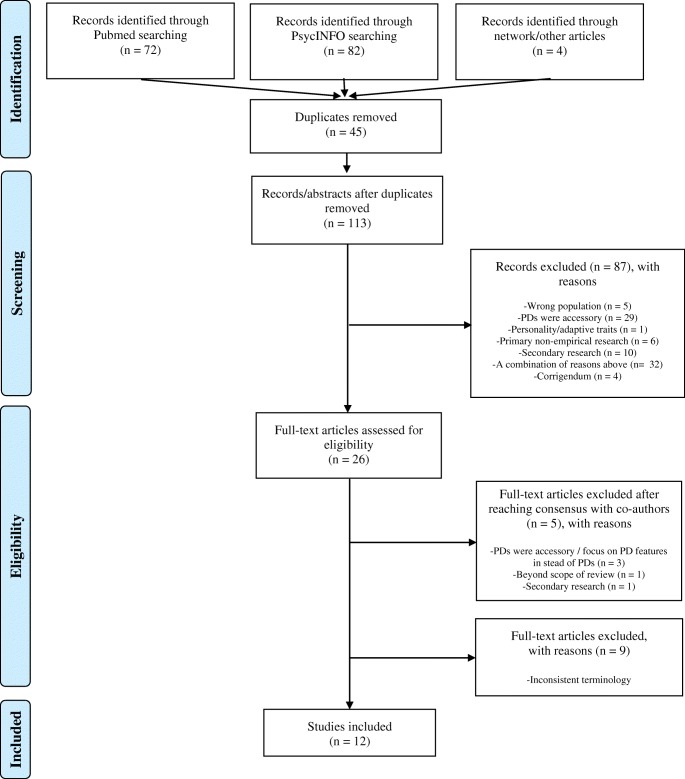
Flowchart review process

The search consisted of the keywords “personality disorder(s)” or “personality pathology” and “older adults” or “elderly” or “late(r) life”, which were required to appear in the article title or abstract. English and Dutch articles of primary empirical studies were included when PDs (conform DSM) in adults aged 60 years and older were the primary focus, and the studies addressed PDs in relation to epidemiology, assessment, or treatment. Articles were excluded if (1) PDs were accessory (e.g., covariate or comorbid condition) or when (2) they addressed personality or (adaptive) personality traits; (3) other than DSM, typologies (e.g., “type A”) were used; (4) the main focus was on adults < 60 years; (5) they addressed themes beyond the scope of this review; and (6) it contains primary non-empirical or secondary research (e.g., case-studies, Delphi-studies, and reviews). The abstracts and articles were reviewed by the first author. In the case of doubt, the other authors reviewed the concerning abstracts and/or articles as well. Then, in- or exclusion followed when consensus (agreement between at least 3 authors) was reached. Figure 1 describes the selection process in detail.

## Results

### Epidemiology: Prevalence and Associations With Cognitive Disorders and Quality of Life

In total, 5 studies reported prevalence rates of PDs in older adults; one of these studies also addressed the association with cognitive disorders and another examined health-related quality of life in the presence of PDs as well. Table 1 summarizes the reported prevalence rates across studies.

**Table 1:** Summary of PD prevalence across studies

Author	Population	Instrument	Prevalence (rounded %)				
Holzer et al. [6]	Community-dwelling adults aged 50 + years in the USA 2001–2002 National Epidemiologic Survey on Alcohol and Related Conditions Study (*N* = 16,884)	Alcohol use disorder and associated disabilities interview Schedule-DSM-IV version (AUDADIS-IV)	General PD= 10.6				
			Obs.-comp. PD= 6.5				
			Paranoid PD= 2.5				
			Schizoid PD= 2.4				
			Antisocial PD= 1.7				
			Avoidant PD= 1.4				
			Histrionic PD= 0.8				
			Dependent PD= 0.4				
Reynolds et al. [7]	Community-dwelling adults aged 50 + years in the USA 2004–2005 National Epidemiologic Survey on Alcohol and Related Conditions Study (*N* = 12,312; 55–64 years *N* = 5,135, 65–74 years *N* = 3,634, 75–84 years *N* = 2,673 & 85 + years *N* = 870)	Alcohol use disorder and associated disabilities interview Schedule-DSM-IV version (AUDADIS-IV)	Total (55-85+ years)	Young-old (55–64 years)	Middle-old (65–74 years)	Old-Old (75–84 years)	Oldest-old (85 + years)
			General PD= 14.5	General PD= 18.1	General PD= 13.2	General PD= 10.4	General PD= 10.7
			Obs.-comp. PD= 6.5	Obs.-comp. PD= 7.6	Obs.-comp. PD= 6.3	Obs.-comp. PD= 4.9	Obs.-comp. PD= 5.4
			Narcissistic PD= 3.9	Narcissistic PD= 4.9	Narcissistic PD= 3.4	Narcissistic PD= 3.0	Narcissistic PD= 2.6
			Borderline PD= 3.2	Borderline PD= 4.7	Borderline PD= 2.5	Schizoid PD= 1.8	Paranoid PD= 1.3
			Schizotypal PD= 2.4	Schizotypal PD= 3.5	Paranoid PD= 2.0	Schizotypal PD= 1.7	Borderline PD= 1.2
			Paranoid PD= 2.3	Paranoid PD= 3.2	Schizoid PD= 1.8	Borderline PD= 1.6	Schizoid PD= 1.1
			Schizoid PD= 2.2	Schizoid PD= 2.8	Schizotypal PD= 1.5	Paranoid PD= 1.2	Schizotypal PD= 1.1
			Antisocial PD= 1.6	Antisocial PD= 2.6	Antisocial PD= 1.3	Antisocial PD= 0.6	Avoidant PD= 0.9
			Avoidant PD= 1.3	Avoidant PD= 2.0	Avoidant PD= 0.9	Avoidant PD= 0.6	Histrionic PD= 0.6
			Histrionic PD= 0.7	Histrionic PD= 0.9	Histrionic PD= 0.7	Histrionic PD= 0.4	Dependent PD= 0.2
			Dependent PD= 0.3	Dependent PD= 0.4	Dependent PD= 0.1	Dependent PD= 0.2	Antisocial PD= 0.1
Pilleron et al. [9]	Community-dwelling adults aged 65 + years in Central Africa 2011–2012 (*N* = 1, 772; Central African Republic *N* = 860, Republic of Congo *N* = 912))	Personality Diagnostic Questionnaire-4+ (PDQ-4+), only the dependent personality domain.	Total	Normal cognitive status			
			Dependent PD= 14.5	Dependent PD= 12.7			
			Central African Rep.	Mild Cognitive Impairment			
			Dependent PD= 17.5	Dependent PD= 26.5			
			Rep. of Congo	Dementia			
			Dependent PD= 11.7	Dependent PD= 25.8			
Oltmanns et al. [8]	Community-dwelling adults aged 55–64 years in the St. Louise area in the USA (*N* = 1,630)	Structured interview for DSM-IV Personality (SIDP-IV)	Clinical interview (SIDP-IV)	Questionnaire self-report (MAPP)	Questionnaire informant-report (MAPP)		
			Sub-thres. PD= 9.3	Obs.-comp. PD= 5.0	Obs.-comp. PD= 12.3		
			At least 1 PD= 8.2	Schizoid PD= 2.5	Schizoid PD= 4.6		
			2 or 3 PDs= 1.0	Avoidant PD= 1.7	Avoidant PD= 1.7		
			PD NOS= 1.8	Paranoid PD= 1.2	Paranoid PD= 3.7		
		The Multisource Assessment of Personality Pathology (MAPP); administrated to both participants and informants	Obs.-comp. PD= 2.9	Antisocial PD= 0.6	Antisocial PD= 3.5		
			Avoidant PD= 2.5	Histrionic PD= 0.6	Histrionic PD= 1.2		
			Narcissistic PD= 1.2	Schizotypal PD= 0.5	Schizotypal PD= 0.8		
			Paranoid PD= 0.8	Narcissistic PD= 0.3	Narcissistic PD= 3.3		
			Schizoid PD= 0.7	Borderline PD= 0.3	Borderline PD= 1.5		
			Antisocial PD= 0.6	Dependent PD= 0.1	Dependent PD= 0.8		
			Borderline PD= 0.4				
			Histrionic PD= 0.2				
			Dependent PD= 0.1				
			Schizotypal PD= 0.1				
Courtois et al. [12]	Adults aged 55 + years living in nursing centers or senior citizen clubs in France (*N* = 83)	Questionnaire on Personality Traits- French Version (QPT; equivalent of the International Personality Disorders Examination)	General PD= 57.8				
			Avoidant PD= 20.5				
			Obs.-comp. PD= 12.1				
			Paranoid PD= 12.1				
			Dependent PD= 3.6				
			Borderline PD= 3.6				
			Schizoid PD= 2.4				
			Narcissistic PD= 2.4				
			Schizotypal PD= 1.2				
			Antisocial PD= 0.0				
			Histrionic PD= 0.0				

The largest study [6], based on the National Epidemiologic Survey on Alcohol and Related Conditions (NESARC) using Wave 1 data of 16,884 community-dwelling adults aged 50 and older, revealed a prevalence rate of roughly 10.7% for having at least one PD. The highest rates were found for obsessive-compulsive PD (6.5%) followed by paranoid PD (2.5%), while dependent PD (0.4%) and histrionic PD (0.8%) were the least prevalent. Furthermore, the individuals with PDs were more likely to be male, younger, and higher educated than the individuals without PDs. However, this study did not address the whole PD range; borderline, schizotypal, and narcissistic PDs were not assessed.

Using Wave 2 data of NESARC, which covered all ten PDs, Reynolds and colleagues [7] found that the prevalence of PDs among older adults is approximately 14.5%. Again, obsessive-compulsive PD was the most prevalent (7.6%), now followed by narcissistic PD (3.9%). Once more, dependent PD (0.26%) and histrionic PD (0.70%) were the least common PDs. The higher prevalence rate might be explained by the inclusion of all the 10 PDs, as borderline (3.2%) narcissistic, and schizotypal (2.4%) PDs which were absent in Wave 1 data appeared to be quite prevalent in this study. Furthermore, PDs were significantly more common in older men (16.8%) than in elderly woman (12.7%), with the exception of paranoid, avoidant, and dependent PD which were more prevalent in females. They also differentiated the prevalence rate within four age groups: young-old, middle-old, old-old, and oldest-old. Aside from dependent PD, all PDs differed significantly across age-groups. Overall, with increasing age, prevalence rates decreased. However, for avoidant, obsessive-compulsive, paranoid, and histrionic PD, a slight but non-significant upturn in occurrence was noted from the old-old to the oldest-old. This was mainly due to increasing prevalence rates for these PDs among men, except for obsessive-compulsive PD which rose with almost 35% in the most aged women.

The study of Oltmanns and colleagues [8••] using data of 1630 middle-aged community-dwelling adults (55–65 years) of the St. Louis Personality and Aging Network (SPAN) revealed a prevalence rate of about 11%; 8.2% had at least 1 PD, 1.8% with the criteria of personality disorder not otherwise specified (PDNOS), and roughly 1% qualified for having two or three PDs. In line with the other studies, obsessive-compulsive PD was most frequently observed (2.9%) now followed by avoidant PD (2.5%). Schizotypal (0.1%), dependent (0.1), and histrionic PD (0.2%) were the least common. Additionally, they found that 9.3% of the participants fell one criterion short of a diagnosis, and could therefore be classified as having a “subthreshold” PD. When comparing these findings to those of Reynolds and colleagues’ [7] subsample of young-old adults, the prevalence rates in the latter study are substantially higher with the exception of avoidant PD and even remain higher when the PD rates in Oltmanns’ study are combined with the subthreshold rates. As both research groups used seemingly comparable samples, namely community-dwelling adults aged 55–64 years living in the USA, and the assessment of PDs differed, this might explain these deviating PD rates.

Although the findings from NESARC and SPAN studies showed that dependent PD is quite infrequently observed (0.1%–0.3%) this PD appeared to be substantially more prevalent in Central Africa. In a cross-sectional multicenter community-dwelling population in Central Africa (*n* = 1772), the prevalence of dependent PD was 14.4% [9]. Moreover, the prevalence rates differed depending on cognitive status. The highest rates were found among the elderly with MCI (26.5%), followed by the elderly with dementia (25.8%). In older adults without cognitive disorders, the prevalence rate was 12.7%. Pilleron and colleagues attributed their surprisingly high prevalence rate to the use of DSM-IV criteria [4] which are insufficiently adapted to older adults, as well as to the older adults’ proneness to respond confirmatory on PD items due to physical illnesses rather than psychological disturbances. Although these factors are known to complicate the assessment of PDs within the aged, they seem unlikely to explain the discrepancy in prevalence as the NESARC and SPAN studies (and nearly all studies for that matter) rely on instruments based on DSM PD criteria as well. Furthermore, these studies should encompass this “proneness” at least to a certain extent as well, as they all sampled older adults. It seems more likely that the use of the Personality Disorders Questionnaire (PDQ-4), which has a high false positive rate [10] and cultural aspects might account for this difference, as studies show that individuals living in more traditional societies, as is Central Africa, score higher on dependent traits than those in more westernized cultures [11]. Moreover, the cognitive status might bias the judgements about PDs; interviewees might have attributed some items (incorrectly) to enduring personality patterns instead of to the person’s current (cognitive) state. In some cases, informants even responded to the PD questionnaire on behalf of the older adult, which might introduce bias too.

One study examined PD prevalence in a non-community-dwelling elderly population. In a sample of 83 older adults living in nursing centers or joining senior citizen clubs, about 58% were diagnosed with a PD when using the French version of the Questionnaire on Personality Traits [12]. Avoidant (20.5%), obsessive-compulsive (12.0%), and paranoid PD (12.0%) appeared to be the most prevalent while both histrionic and antisocial PD were absent in this sample. All PDs were more prevalent in men, except schizotypal PD which was more often seen in women than men (1.7% versus 0.0%). However, some caution is warranted when interpreting the numbers, as the sample size is rather small, it relied on self-report which might be biased due to interference of age-related cognitive decline, and the questionnaire on personality traits (QPT) tends to overestimate the prevalence PDs [13].

Aside from prevalence rates, Pilleron and colleagues [9] examined the relationship between dependent PD and cognitive disorders. They found that after controlling for sociodemographic, vascular, and psychological covariates, the elderly with dependent PD were 2.18 times more likely to have MCI, than the older adults without this PD. Although dependent PD was also associated with dementia after adjusting for sociodemographic factors (OR = 1.98, CI = 1.25–3.14, *ρ* = 0.004), this association faded when vascular and psychological factors were taken into account.

Holzer, Huang [6] investigated the association between PDs and physical health-related quality of life (PHRQoL). They found that the presence of a PD was related to clinically significant worse PHRQoL compared with older adults without these disorders. However, after taken sociodemographic and psychosocial covariates into account, only dependent, paranoid, and obsessive-compulsive PDs remained associated with decreased PHRQoL. These findings suggest that specific symptoms of these PDs may be related to PHRQoL. Still, it is also possible that that an unmeasured covariate may be present, given that PD diagnoses combined with sociodemographic factors only explain 15% of the variance of PHRQoL score.

As these 5 studies used a variety of research methods and PD measures, direct comparison between prevalence rates is complex. Most of these instruments, although validated in various settings, have not yet been corroborated in older adults. Furthermore, these are based on the DSM criteria which contain measurement bias in older adults [3, 14]. Different sample types (community-dwelling, non-community-dwelling) with varying comorbidity and differences in age distribution across samples as well as diverse cultures (USA, Europe, Africa) may affect prevalence rates as well.

### Clinical Implications

These studies prove that PDs are quite prevalent in later life [6–9, 12], and as they are known to have a serious impact on one’s life, such as high levels of suffering and decreased functioning [15], straining the clinician-patient relationship by (interpersonal) difficulties, treatment rejection, and non-compliance [15], it is important for clinicians to be alert to PDs in older adults.

Moreover, the presences of a PD may complicate the recognition of/and adversely affect treatment of comorbid disorders [16]. In the case of PDs, treatment outcomes are poor(er), whereas the rates of relapse and readmission increases [17]. This, in addition to the already complex clinical picture of older adults who often have multiple medical conditions, whether or not combined with psychiatric comorbidity and polypharmacy [18], seems to make it all the more important to have valid and reliable instruments to facilitate “early” detection of PDs in older adults [19]. Being more attentive to PDs in late life may enable the clinician to take personality functioning into account when considering his approach and communication to optimize older adults’ treatment compliance and to tailor interventions to accommodate the specific needs of individual patients [20] which may enhance the odds of a positive response to treatment and circumvent treatment dropout. Furthermore, it may allow the clinician (such as general practitioners, geriatricians) to screen for PDs and to make faster and more specific referrals to mental health settings where further diagnostic assessments and treatment options are available [20].

### Assessment

Diagnosing PDs, which ideally requires the use of multiple sources of information, is a challenging endeavor due to the complex and multifaceted nature of these disorders. This is even more true when assessing PDs in older adults where multimorbidity obscuring the clinical picture is the rule rather than the exception, and available instruments, mostly designed for (young) adults, are not automatically applicable to the elderly [19]. This severely complicates the already complex diagnostic process. Hence, there is a need for age-appropriate instruments.

Since 2015, 8 studies addressed the assessment of PDs in older adults, mostly by validating existing instruments within older adults or by examining their age-neutrality. The investigated instruments parallel the full PD spectrum of DSM-5; the studies used the Gerontological Personality disorders Scale (GPS; [21]) which is based on section II general PD criteria, the Structured Interview for DSM-IV Personality (SIDP-IV; [22]), the Questionnaire in Personality traits (QTP/VKP-4; [23]), the Multisource Assessment of Personality Pathology (MAPP; [24]), and the Assessment of DSM-IV Personality Disorders (ADP-IV; [25]) all addressing section II specific PD criteria. To overcome the issues linked to categorical classification, such as diagnostic heterogeneity within categories and extensive co-occurrence of PDs, a dimensional counterpart, the alternative model for personality, was included in the DSM-5 section III “emerging measures and models” which stimulated research in the elderly as well. The Severity Indices of Personality Problems Short Form (SIPP-SF, derived from the SIPP-118; [26]) which covers criterion A level of personality functioning and the Personality Inventory for DSM-5 Brief Form [27] tapping onto the trait dimensions of criterion B were both validated as well as investigated with regard to their age-neutrality. The latter analysis was also done for the Dimensional Assessment of Personality Pathology Basic Questionnaire (DAPP-BQ) which measures maladaptive trait dimensions. Table 2 summarizes the main findings per study.

**Table 2:** Summary of studies addressing the validity of PD assessment in older adults

Author	Population(s)	Personality instrument(s)	Type of instrument	Significant validity findings
Debast et al. [28•]	Dutch-speaking community-dwelling adults aged 61–99 years (*N* = 171)	Focal instrument(s)		SIPP-SF
		Severity Indices of Personality Problems-Short Form (SIPP-SF; Responsibility was excluded from all analyses)	Self-report questionnaire measuring DSM-5 section III criterion A personality functioning	Cross-age validity
				94% age-neutral on item level
				75% age-neutral on scale level
	Geropsychiatric inpatient adults aged 60–81 years in Flanders, Belgium (*N* = 59)			Limited or no age differences in mean scores
		Personality Inventory for DSM-5-Brief Form (PID-5-BF; Negative Affectivity and Antagonism were excluded from DIF/DTF analyses, Antagonism was excluded from construct validity analyses)	Self-report questionnaire measuring DSM-5 section III criterion B personality traits	Known-group/discriminative validity
				Identity Integration and Self-Control differentiate patients from nonpatients
	Dutch-speaking community-dwelling college students aged 17–31 years (*N* = 210)			
				Convergent validity in geropsychiatric inpatients
				Significantly correlated to GPS total
				Significantly correlated to GPS BIO, except for social concordance
		Secondary instrument(s)		
		Gerontological Personality disorders Scale-patient version GPS-pv;GPS HAB was excluded from all analyses	Self-report questionnaire measuring DSM-5 section II general PD criteria	PID-5-BF
				Cross-age validity
				75% age-neutral on item level
				0% age-neutral on scale level
				Known-group/discriminative validity
				Negative affectivity, detachment, disinhibition, and psychoticism differentiate patients from nonpatients
				Convergent validity in geropsychiatric inpatients
				Negative affectivity, disinhibition, and psychoticism were significantly correlated to both GPS total and GPS BIO
				SIPP-SF & PID-5-BF
				Convergent validity
				Significant correlations between all SIPP-SF & PID-5-BF domains in community-dwelling older adults
				Correlations between SIPP-SF & PID-5-BF were larger for older adults than young adults
Debast et al. [30]	Dutch-speaking community-dwelling adults aged 65–99 years (*N* = 293)	Focal instrument(s):		Concurrent validity
		Personality Inventory for DSM-5- Brief Form (PID-5-BF)	Self-report questionnaire measuring section III criterion B personality traits	Significant positive correlations with PID-5
				Factorial/structural validity
				Negative Affectivity, Detachment, Antagonism, Disinhibition and Psychoticism were corroborated as higher order domains
		Secondary instrument(s)		
				Convergent and discriminant validity
		Personality Inventory for DSM-5 (PID-5)	Self-report questionnaire measuring section III criterion B personality traits	
				Significant positive correlations with GPS total score, GPS HAB and GPS BIO
				Significant negative correlations with SIPP-SF
		Severity Indices of Personality Problems-Short Form (SIPP-SF)	Self-repo0rt questionnaire measuring DSM-5 section III criterion A personality functioning	
		Gerontological Personality disorders Scale-patient	Self-report questionnaire measuring DSM-5 section II general PD criteria	
Rossi et al. [29]	Dutch-speaking community-dwelling college students aged 17–31 years (*N* = 210)	Focal instrument(s):		Cross-age validity
		Severity Indices of Personality Problems-Short Form (SIPP-SF)	Self-report questionnaire measuring DSM-5 section III criterion A personality functioning	Limited age differences in mean scores on all domains, except for responsibility which shows medium age differences
				Medium to large age differences in partial correlations/convergent/divergent validity between personality fucnctioning (SIPP-SF) and pathological personality traits (PID-5 and DAPP-BQ), with higher correlations for the older adults.
				Factorial/structural validity
				Identity integration, relational capacities, responsibility, self-control and social concordance were corroborated as higher order domains
				Convergent and discriminant validity
				Significant medium to large positive correlations between all SIPP-SF and all PID-5 domains
				Significant medium to large positive correlations between most SIPP-SF and DAPP-BQ domains
	Dutch-speaking community-dwelling adults aged 61–99 years (*N* = 171)	Secondary instrument(s):		
		Personality Inventory of DSM-5 (PID-5)	Self-report questionnaire measuring section III criterion B personality traits	
		Dimensional Assessment of Personality Pathology –Basic Questionnaire (DAPP-BQ)	Self-report questionnaire measuring section III criterion B personality traits	
Penders et al. [20]	Community-dwelling adults aged 60-91 years in the Netherlands (*N* = 302)	Focal instrument(s):		GPS-PV:
		The Gerontological Personality disorders Scale-patient version (GPS-pv)	Self-report questionnaire measuring DSM-5 section II general PD criteria	Diagnostic accuracy
				No optimal cut-off score for discriminating between the presence and absence of PDs
	Community-dwelling informants aged 31–89 years in the Netherlands (*N* = 302).	The Gerontological Personality disorders Scale-informant version (GPS-iv)	Informant-report questionnaire measuring DSM-5 section II general PD criteria	GPS-IV
				Diagnostic accuracy
				≥ 3 is optimal cut-off score for discriminating between the presence and absence of PDs; sensitivity = 78% and specificity = 65%
		Secondary instrument(s)		
		The Informant Personality Questionnaire (HAP)	Informant-report questionnaire measuring premorbide personality traits linking to DSM-5 section II specific PD criteria	
Debast et al. [32]	Inpatient (for alcohol and drug treatment) adults aged 18–75 years in Flanders, Belgium (*N* = 312; younger adults 18–34 years *N* = 107; middle-aged adults 35–59 years *N* = 107; older adults 60–75 years *N* = 107)	Focal instrument(s):		Cross-age validity
		The Assessment of DSM Personaity disorders-IV (ADP-IV; used both categorically and dimensionally)	Self-report questionnaire measuring DSM-5 section II specific PD criteria	97% age-neutral on categorical item level
				100% age-neutral on categorical scale level
				95% age-neutral on dimensional item level
				100% age-neutral in dimensional scale level
				Significant age differences in mean scores on 8 of 10 PDs; overall older adults scored lower than young adults
		Secondary instrument(s):		
		-		No age differences in mean scores between older adults and middle-aged adults
Oltmanns et al. [8]	Community-dwelling adults aged 55–64 years in the St. Louise area in the USA (semi-structured diagnostic interview administered to *N* = 1,630, self-report filled out by *N* = 1,608)	Focal instrument(s):	Interview measuring DSM-5 section II specific PD criteria	Convergent validity
		The Structured Interview for DSM-IV Personality (SIDP-IV)		Low to moderate correlations between SIDP, self-MAPP, and informant-MAPP
				Highest correlations between SIDP and self-MAPP, whereas the correlations between SIDP and informant-MAPP were the lowest
		Self-report Multisource Assessment of Personality Pathology (self-MAPP)	Self-report questionnaire measuring DSM-5 section II specific PD criteria	
				Highest level of concordance among the three sources on avoidant and borderline PD
		Informant –report Multisource Assessment of Personality Pathology (informant-MAPP)	Informant-report questionnaire measuring DSM-5 section II specific PD criteria	Informant-MAPP was less conservative than SIPD and self-MAP, except for avoidant PD
	Community-dwelling informants aged 55-64 years in the St. Louise area in the USA (*N* = 1, 447)			
		Secondary instrument(s):		
		-		
Courtois et al. [12]	French-speaking community-dwelling psychology students aged 18–41 years (*N* = 158)	Focal instrument(s):		Convergent validity
		Questionnaire on Personality Traits (QPT)-French version, equivalent of the International Personality Disorders Examination	Self-report questionnaire measuring DSM-5 section II specific PD criteria	Schizoid PD was negatively correlated to agreeableness
				Antisocial PD was negatively correlated to agreeableness
				Borderline PD was negatively correlated to openness while positively correlated to Neuroticism
		Secondary instrument(s):	Self-report questionnaire measuring personality traits	Histrionic PD was positively correlated to both agreeableness and conscientiousness
		The Big Five (BIG-5), French-version		
				Narcisisstic PD was negatively correlated to neuroticism
	Adults aged 55 + years living in nursing centers or senior citizen clubs in France (*N* = 83)			Avoidant PD was negatively correlated to both openness and extraversion
				Dependent PD was negatively correlated to openness, conscientiousness, and extraversion, while positively correlated to neuroticism
				Obsessive-compulsive PD was negatively correlated to both conscientiousness and agreeableness

In the past years, three personality measures have been examined regarding their construct validity in older adults. These were all relatively short instruments, mainly relying on self-report, the SIPP-SF [28•, 29], the Personality Inventory for Diagnostic and Statistical Manual of Mental Disorders, Fifth edition, Brief Form (PID-5-BF) [28•, 30], and the GPS [20, 31]. The 60-item version of the SIPP was significantly correlated to other PD instruments (i.e., the GPS, the PID-5-BF, and the DAPP-BQ) [28•, 29] and was to be able to differentiate between older adults with normal personality functioning and those with personality dysfunctioning [28•]. Beside construct validity, the concurrent validity of the PID-5-BF in older adults was established as well. This brief version, consisting of 25 items, was significantly positively correlated to the 220 items of the original PID-5, and the same higher order domains were corroborated [30]. Furthermore, the PID-5-BF proved to be able to discriminate older adults with maladaptive personality traits from the elderly with adaptive personality traits [28•]. These short tests appeared to be useful for assessing personality functioning and maladaptive personality traits, which taken together form the basis of the DSM-5’s alternative model of personality disorders [5]. Lastly, both self- and informant-report versions of the Gerontological Personality disorders Scale (GPS; 21) which is a 16-item age-specific screener for PDs among older outpatients in mental healthcare have been validated in community-dwelling older adults sampled from 5 general practices. The sensitivity (78%) and specificity (65%) of the informant version have been found to be adequate. However, the diagnostic accuracy of the self-report version was limited [20]. Aside from its validity, the feasibility and acceptability of the GPS was examined as well [31]. The GPS appeared to be a useful tool in general practice as it was judged to be feasible and acceptable by professionals and older adults and informants.

In addition, since 2015 a number of studies have examined the cross-age validity of personality measures. Aside from construct validity, the SIPP-SF and the PID-5-BF have been examined for age-neutrality [28•, 29] as has the ADP-IV [32], by comparing mean scores across age groups, differential items functioning (DIF), and differential test functioning (DTF). When an item has DIF, this implies that the probability of endorsing an item response is not the same across two groups (e.g., old and young), even when they display the same level on a common latent personality dimension. The aggregated impact op DIF may jeopardize the comparability of the scale scores across the perspectives, which is assessed with DTF. In the ADP-IV, when used categorically, only 2 of the 79 (2.5%) items assessing personality disorder symptoms had DIF in favor of older adults. When used dimensionally, 4 items were not age-neutral. The amount of DIF, however, did not result in the whole PD scale scores being biased. The mean scores on eight of the ten PDs displayed significant age differences; overall older adults scored lower than young adults. However, there were no age differences between older adults and middle-aged adults. [32]. Personality functioning as measured with the SIPP-IV displayed somewhat more DIF; 3 of the 60 items were biased favoring one age-group over the other. On a scale level, relational capacities showed DTF [28•]. Furthermore, there were limited to no age differences in mean scores [28•, 29]. A more cautious approach is needed when assessing maladaptive traits in older adults with the PID-5-BF, as 25% of the items and all domains appeared to function differently across age [28•].

Courtois and colleagues did an exploratory study toward the development of the French version of the QPT by assessing its association with the Big Five Inventory. They found that PDs in older adults as assessed with the QPT were significantly correlated with Openness, Conscientiousness, Extraversion, Agreeableness, and Neuroticism [12].

As assessment of PDs should ideally involve multiple sources, questions emerge regarding agreement and which source indicates more pathology. Oltmanns and colleagues [8••] compared three sources of information in a sample of older adults; a clinical interview (SIDP-IV), a self-report questionnaire, and an informant-report questionnaire (both MAPP). They found that the concordance between the measures was low to moderate, indicating that they identify some of the same symptoms but mainly provide unique information. Unsurprisingly, the agreement between the SIDP-IV and the self-report MAPP was the highest, which is probably due to shared variance as they both tap into the self-report perspective. Further, the clinical interview appears to be the most conservative in assessing PDs in older adults, followed by self-report. Informants, then again, reported the most pathology.

Current research revealed that the SIPP-SF, the PID-5-BF, the DAPP-BQ, and the GPS are adequate instruments for assessing PDs in older adults. However, as the studies were conducted in either general population or highly specific and relatively small clinical populations, the generalizability of these results may be limited. Overall, these studies pointed out that the expressions of personality functioning (criterion A) can be more reliably compared and assessed over age-groups than maladaptive personality traits (criterion B) can. Furthermore, it appeared that personality functioning and pathological traits were more and strongly correlated in older adults than in young adults, suggesting that in older adults certain domains of personality functioning can be more indicative for the presence of maladaptive traits. Including other sources of information, such as informant-report, can aid in the assessment of PDs. However, the choice of source can impact the findings as they may vary in the level of conservativeness.

### Clinical Implications

In comparison to adults (< 60 years), there are only few reliable and valid instruments for diagnosing PDs in older adults. Aside from the lack of age-adjustments, many PD self-report questionnaires and semi-structured interviews rely on a large number of complex items (e.g. abstract language use, use of double negatives), which may often be quite burdensome for older adults. Fortunately, research on assessing PD in older adults is growing and recent efforts show that there are now a number of brief, age-neutral, and valid (although mostly self-report) instruments available, albeit for general or highly specific populations [8••, 20, 28•, 29–32].

However, it is important to also use other sources of information in complementing PD assessment as well as guiding its interpretation [33]. Older adults might have had several (psychological) treatments during their life, wherewithal medical and/or psychiatric records [34]. Biographical information and informant information might be available as well which could provide insight regarding personality traits and psychosocial (dys)functioning. This information can shed some light on the enduringness of personality pathology. Informant report may prove useful when verifying life-events, overcoming the possible effects of sensory and cognitive impairment troubling self-report. Additionally, it may also have incremental value by adding crucial information in PD assessment, as self-report might be biased due to the presence of severe psychopathology, limited self-awareness, distorted self-perceptions, or as a result of reluctance to disclose problems [35–37]. Furthermore, as the clinical presentation of PDs in late life might be more complex due to cognitive aging, psychopathology, medical conditions, and polypharmacy [18], including various sources of information to the assessment of PD, if needed complemented by medical examination (e.g. excluding medical conditions such as head trauma), is therefore recommendable.

### Treatment

Since 2015, only one study has been published examining PD treatment effectiveness in later life. This study used a non-concurrent multiple-baseline design to investigate the value of schema therapy in eight elderly mental health outpatients (62–76 years) with a primary diagnosis of a cluster C PD or PDNOS with cluster C traits as assessed by the Dutch version of the Structured Clinical Interview for DSM-IV PDs (SCID-II; [38]). Videler and colleagues [39••] found that schema therapy decreased the credibility of dysfunctional core belief, led to symptom and complaint reduction, and improved the quality of life. At follow-up, the participants did no longer meet the general or cluster-specific criteria of a PD, and they were remitted from PD diagnosis. Moreover, most of the effect sizes of these findings were high, which is in line treatment effects in (young) adults [40].

Although this showed that schema therapy is feasible and effective in later life PDs, results cannot be merely generalized to other populations or other PDs, given the small sample size and its focus on cluster C PDs only.

### Clinical Implications

The current search pointed out that treatment of PDs in older adults is still a highly underexplored topic, probably stemming from a therapeutic nihilism. However, the study of Videler and colleagues [39••] showed that treating PD in older adults can be effective and feasible, as do the findings of a small number of (case) studies [41–43], thereby pointing out that this skepticism is unwarranted.

When deciding on the most appropriate intervention for an older adult with personality pathology, it is important to not only evaluate his/her needs, level of motivation and cooperation, type and severity of the PD, but to also take the degree of functional limitations reflecting somatic and cognitive comorbidity into account [44, 45]. Besides, specific gerontological treatment concerns should be essential topics in treatment, such as beliefs about and the consequences of somatic ailments, changing life perspective, cohort and sociocultural context-bound beliefs, intergenerational linkages, and the loss of social roles [42]. Treating PDs in older adults therefore seems to require a more tailored and personalized approach.

Furthermore, experts believe that interventions for PDs in older adults can be considered allocated on a continuum, ranging from supportive-structuring treatment approaches (e.g., psychoeducation, behavioral counseling) to adaptation-focused (e.g., social skills training) to personality-changing interventions (e.g., schema therapy, dialectical behavior therapy) for each of which in- and exclusion criteria were formulated [44, 45]. Moreover, initial results show that applying clinical staging of PDs in treatment indication might improve its outcomes and may facilitate early detection [46, 47]. While empirical research addressing interventions for older adults with PDs are needed, the above-described recommendations can aid in defining achievable treatment goals and choosing the most appropriate treatment level.

## Discussion

While reviewing the eligibility of the articles addressing epidemiological, assessment, and treatment aspects of PD in older adults (Fig. 1), there appeared to be an inconsistency of terminology. Although in the first instance all the articles seemed to be focused on PDs, a considerable number of articles (i.e., 9) turned out to be less clear about their central theme, thereby excluded from the current review. The terms PDs, PD features, PD symptomatology, and (high levels of) PD symptoms were used interchangeably. Furthermore, whereas a number of studies gave the impression to concentrate on PDs, after carefully reading only a minority of the participants met the full criteria of PDs. In other studies, the prevalence of PDs was absent, or the information published did not permit the identification of participants with PDs.

With about 35% of the potentially eligible articles using inconsistent terminology, it seems that the construct and/or definition of PDs in older adults might be not that clear or straightforward. Factors like different and more atypical clinical presentations in later life, a more temporal instable course, and late(r) onset [48, 49] may have contributed to this. This has multiple implications. On a more practical level, it complicates comparability between studies, impeding generalizability of the findings which are already scarce in this age group. On a fundamental level, it interferes the diagnostic process which, especially in older adults, is already no *sinecure*. Consequently, as diagnosis precedes and guides interventions, it hampers treatment as well.

The inconsistency in terminology might also signal the importance of examining the full spectrum of personality functioning and traits. Indeed, the distinction between the presence and absence of a PD is somewhat arbitrary. With lowering by 1 criterion, the prevalence of PDs in older adults would almost double [8••]. Furthermore, a substantial number of people exhibit at least some symptoms of personality pathology. Aside from investigating full-blown PDs, further research on PD symptoms in older adults is relevant as studies show that these symptoms may be vital in predicting physical health problems and other mental disorders [15, 50, 51].

Reviewing recent studies reveal that promising steps are taken in the field of late life PD. However, further PD research, irrespective of its theme, should be carried out among the full range of older adults, such as investigating the previous examined instruments across various settings and cultures of older adults. Furthermore, studies developing age-specific cutoff scores for domains displaying DIF (and not DTF which implies that it does not represent the same measurement across groups), establishing age-related norms, and developing informant-report instruments are needed. Moreover, it would be valuable to examine which content is best assessed by older adult self-report and what kind of information should be captured from an informant perspective. Also replications of Videler’s treatment study [39••], preferably RCTs, with larger sample sizes and addressing the whole PD spectrum are needed. Exploring the effectivity of other treatment modalities for PDs in older adults, albeit personality changing, adaptation enhancing, or contextually focused, is of importance as well including empirical studies addressing the usefulness of in- and exclusion criteria per treatment level and clinical staging and health management of PDs.

### New Areas of Interest

Because of the global aging population, increasing clinical and scientific attention for PDs in older adults is of importance, and also new areas of interest such as other settings, and behavioral counseling may arise.

Recent PD research has been mainly focused on older adults from either general or highly specific inpatient populations. Although research within these populations should be stimulated, other settings, such as assisted living facilities, geriatric medicine, and general practice, should be considered as well.

Considering the prevalence of PDs in older adults, the aging population, and their central role in healthcare, general practitioners (GPs) are often the first and perhaps the most frequently contacted healthcare provider, who is consulted by older adults with PDs, although these disorders, given their ego-syntonic nature, are usually not the main reason for consultation. Therefore, GPs can play a key role in the detection of PDs in older adults. Early detection is important as it may circumvent certain negative consequences of PDs and contribute in reducing economic costs, by making referrals to specialized mental health care for further assessment and treatment, preventing hospital admissions, taking personality into account, and tailoring treatments whereby minimizing non-compliance and negative treatment outcomes. Given their often longtime involvement, GPs may also provide crucial additional information about the older adult’s lifespan.

However, resources for recognizing and managing PDs in older adults are scarce within this setting (as is research), complicating the GP’s work. Further research within this setting, given the GP’s central role, is urgently needed.

Another new area of interest might be behavioral counseling. This review points out that research on the effectivity of treatment of PD in older adults is still limited. Research that has been conducted was focused on interventions on (a more direct) patient-level. However, factors like severe cognitive deficits or treatment rejection can severely hamper intervention options in older adults with PD, sometimes making (direct) psychological treatments impossible. In these cases, a more contextual and indirect approach such as behavioral counseling might be an acceptable and appropriate alternative. In behavioral counseling, the intervention is of an indirect nature, as the patient’s behavior is treated by influencing his/her significant others, caretakers, or nursing staff.

A promising form of behavioral counseling, based on the cognitive therapy, is the Cognitive Model for Behavioral Interventions (CoMBI; [52]), which is specifically developed for nursing staff-members dealing with older adults with PDs. In this protocol the ten PDs are described by means of self-image, perception of others, eliciting event, and the patients’ challenging behavior. The patient’s core needs to play a central role in this intervention. This model assumes that when the patient’s core needs are insufficiently met, due to a triggering event, both his/her self-image and perceptions of others will be confirmed thereby provoking the challenging behavior. However, if the triggering event can be substituted by staff members addressing the patient’s core needs, the challenging behavior will decrease. The feasibility of this 4-step protocol is currently investigated in several facilities.

## Conclusions

Since the review of van Alphen and colleagues [2•], only a limited number of studies addressed epidemiological, assessment, and treatment aspects of PDs in older adults. Nonetheless, these studies hold a promising view. Aside from the attempts to map the prevalence of PDs in later life, studies show continuing efforts examining the age-neutrality of existing items and instruments, developing age-specific measures, and validating PD questionnaires in older adults, herewith mainly using instruments tapping into the DSM-5’s alternative model of PDs. Moreover, there is initial proof of treatment effectiveness. These studies show that (research on) PD in older adults should not be dismissed, but that they hold the future.
